# The Impact of COVID-19 on Staff Working Practices in UK Horseracing

**DOI:** 10.3390/ani10112003

**Published:** 2020-10-30

**Authors:** Emma Davies, Will McConn-Palfreyman, Jane M. Williams, Geoff P. Lovell

**Affiliations:** 1 Equine Department, Hartpury University, Gloucester GL193BE, Gloucestershire, UK; jane.williams@hartpury.ac.uk; 2 SportScotland Institute of Sport, University of Stirling, Stirling FK9 4LA, UK; Will.mcconn@gmail.com; 3 Sport Department, Hartpury University, Gloucester GL193BE, Gloucestershire, UK; Geoff.Lovell@hartpury.ac.uk; 4 School of Behavioural and Health Sciences, University of the Sunshine Coast, Sippy Downs QLD 4556, Australia

**Keywords:** horseracing, pandemic, job security, employment, social distancing

## Abstract

**Simple Summary:**

Although coronavirus stopped horseracing in March 2020, most staff were classified as essential workers due to equine care and continued to work throughout lockdown. The physical and psychological impact of working during lockdown is unknown, and staff stress could have negative implications for racehorse welfare. Over half of staff surveyed were still working during lockdown. Racing grooms and stud staff were more likely to be working than most sectors, due to the timing of lockdown with the racing calendar and foaling season. Administrative staff were busier during lockdown, completing additional risk assessments or paperwork. Most staff reported that workplace changes were successful in protecting health and safety, but flat racing grooms felt that work-based changes were less effective. Negative perceptions of work-based COVID-19 changes may affect the staff’s ability to complete daily tasks and thus influence the quality of care provided to horses in their charge. Trainers and part-time staff were concerned about job security, highlighting a need for further employee support structures following the pandemic. The racing industry has prioritised staff health and safety but continued reflection on staff well-being, demands and working practices will maximise staff’s ability to care for horses under their charge, and allow racing to maintain the highest standards of equine welfare.

**Abstract:**

Due to COVID-19, horseracing was required to cease all activity in March 2020; however, little is known about the pandemic’s impact on staff working practices. This study investigated the impact of COVID-19 on staff working practices during the initial lockdown phases. An online survey about working conditions during lockdown was answered by 287 participants. Chi-squared tests for independence and binary logistic regression (BLR) analysis was undertaken. A total of 53.7% (n = 154) of staff were working during lockdown. Pandemic-specific workplace changes were reported as effective by 87.8% (n = 115) of staff. Flat grooms reported workplace changes as less effective (χ^2^ (52, n = 131) = 92.996, *p* < 0.001). A total of 67.2% (n = 193) of staff were positive about job security. Trainers and grooms were significantly less likely to report jobs as secure (χ^2^ (52, n = 287) = 75.653, *p* < 0.05). The findings suggest that most of the racing industry positively received changes made by their employers to tackle the pandemic, and for staff still working during lockdown, their health and safety was prioritised. Continued development of employee support structures to promote job security and workforce stability is advised, which will minimise the disruption of staff changes on the care and welfare of the horses.

## 1. Introduction

Employing over 20,000 people across multiple sectors [1], the UK horseracing industry has been substantially affected by the COVID-19 pandemic and lockdown restrictions implemented on 24 March 2020. Comprised of both flat and jump racing, horseracing is a billion-pound industry, contributing approximately £3.45bn to the UK economy per annum [2]. Most UK flat races are held on grass during the summer racing season (late March–late October), whilst jump racing is traditionally held in the autumn, winter, and spring [3]. The government-imposed lockdown restrictions resulted in the premature end of the 2019/20 jump season and delayed the start of the flat racing season. After agreement from the UK Government, flat racing resumed behind closed doors on 1 June, with jump racing returning on 1 July 2020.

Employment opportunities within horseracing are broad; job demands, working environments and daily working practices may differ depending on the racing sector [4,5,6]. Roles may include racing grooms or trainers, as well as racecourse staff, press and media roles, and national unions and charities [1]. Despite the differences in job roles within the industry, all staff work towards common goals-the advancement of UK racing and the maintenance of high standards of equine welfare. With such an extensive workforce, racing sectors may be experiencing differing work-based challenges during the lockdown period. Anecdotal reports suggest that trainers allocated off-season working activities such as fence painting to ensure job security for grooms, whilst staff from racecourse groups, such as the Arena Racing Company (ARC), were furloughed [7]. Whilst the COVID-19 Steering Group have collaborated across stakeholders to determine best practice for all sectors^1^, little is currently known about how staff are experiencing the pandemic or the effect that the pandemic has had on staff working practices across all sectors. Further exploration of staff perceptions of the pandemic on working life could result in increased awareness of previously unknown work-based stressors, highlight new solutions to support continuing mitigation measures and identify key strategic initiatives for the industry moving forwards.

### 1.1. COVID-19

In mid-December 2019, a novel and contagious atypical viral pneumonia was identified in Wuhan City, Hubei province, China [8]. The virus was identified as primarily targeting the human respiratory system, and in January 2020, researchers isolated a novel severe acute respiratory coronavirus (SARs-CoV-2) [9]. Due to the limited scope available for this paper, readers are directed to other studies for a comprehensive review of symptoms, transmission and mitigation strategies [8,9,10,11,12,13]. Swiftly declared a Public Health Emergency of International Concern, the named COVID-19 virus was updated to a global pandemic by the World Health Organisation (WHO) on 11 March 2020. At the time of writing this manuscript (19 September 2020), 30,700,502 cases of COVID-19 have been identified globally across 213 countries with 956,650 deaths. The first known case of COVID-19 in the UK was late January 2020, with the first death reported on 5 March 2020 [14]. As of the 19 September 2020, a total of 385,936 positive cases and estimated over 50,000 deaths have occurred in the UK [14]. On 24 March 2020, the UK Government enforced a nationwide lockdown, including the cessation of all national and international sport, with individuals advised to only go outside for food, one hour of exercise per day, or work, but only if working from home was not possible [14]. This period of lockdown continued until 13 May 2020, when unlimited exercise was permitted for the general public [14]. On 1 June, sports were permitted to begin play behind closed doors, including the reintroduction of flat racing. At the time of writing this manuscript (19 September 2020), the government has permitted the reopening of non-essential shops, hairdressers, and encouraged most of the UK workforce to return to work where possible. However, the reintroduction of local lockdown enforcement is being utilised due to increasing case numbers in certain areas of the UK [14].

### 1.2. Impact on UK Horseracing

In previous years, the social demand for strict welfare standards in horse racing has led to a culture of ‘putting the horse first’ [15]. These priorities resulted in an industry which has some of the highest welfare standards in the equestrian sector, has continuously worked to promote scientific evidence-based training and welfare principles and upskills staff to maintain those standards [1]. However, the ‘horse-first’ culture may have inadvertently created a workforce who deprioritise their own health and well-being to care for the horse, which has been previously reported in other animal care sectors [16]. Employees who ignore their own health needs may result in higher levels of stress, increasing the risk of occupational injury or disease, impacting the efficiency of a workforce already under high demand [17,18]. Issues of poor horse welfare can arise when training staff are not as engaged and connected emotionally to the horses that they are caring for [19], which may result from a high level of physical and mental fatigue, impacting the ability to maintain the high standards required when working with racehorses.

The racing industry was already experiencing a labour shortage [20], with concerns over retention and recruitment previously high prior to lockdown [5]. Prior to the pandemic, trainers already highlighted that finding staff cover was a main source of stress, in part due to a current staff shortage in racing [5,6,20,21,22], and thus the current circumstances are likely to exacerbate ongoing staffing issues. A recent report suggested that nearly half (46%) of stable staff surveyed are concerned about job security [23]. Although it might be expected that the pandemic would reduce employee working hours, in some professions, including those with a responsibility of care for animals, working hours have remained the same, or even increased [24,25]. It was predicted at the peak of the virus that 10–20% of the population may be suffering due to active illness, recovery or care of another, resulting in significant division of the workforce from work [26], thus increasing work demands on the healthy workforce [25]. Whilst occupational health research often concludes that longer working hours increase stress in employees, wider literature suggests a more nuanced relationship, with stress more likely to occur due to a mismatch in actual and preferred working hours [27,28]. This could suggest that staff on reduced hours due to the pandemic may be at risk of work-related stress due to worries about job security or financial stability, which could be affecting mental health [28]. COVID-19 challenges are predicted to not only have a negative impact on workplace stability, which can affect economic success, staff health and job satisfaction, but COVID-19 challenges may also affect the ability of staff to effectively care of horses on training yards. Racing Welfare saw a substantial influx of new enquiries to their services, which include support on housing, finances, mental health, and employment, with a 2980% increase in new enquires from 17 March (pre-lockdown) (n = 5) to 15 May (n = 154), although the rate of increase has since slowed [29].

Despite specific lockdown guidance, and advisory materials for employees who are unable to work from home, a recent government report suggests that over 1 in 10 (11%) working adults are worried about health and safety in their workplace [14]. An industry report of horseracing grooms found that 36% of yard staff did not feel safe at work during the pandemic. However, this did not consider wider racing sectors who may also have a direct impact on the welfare of the horse, such as stud staff, racing secretaries or sales staff [23]. A review of multiple sectors may present opportunities for the industry to share best practice and identify effective strategies to support staff following lockdown, and longer term during the ongoing pandemic situation. In identifying challenges staff face during the coronavirus pandemic, further action can be taken to support the workforce by employers, or unions and charities such as the National Association of Racing Staff (NARS) or Racing Welfare, thus enabling staff to continue working to high standards and optimising welfare, reducing the risk of injury to horses and humans alike. This study therefore aimed to investigate the impact of COVID-19 on staff working practices across UK horseracing during the initial lockdown phases of 2020. To approach this overarching aim, four specific research objective were addressed: (1) To identify sector specific differences in reported working practices during the pandemic, (2) to determine what workplace health and safety measures horseracing staff report as having been implemented and how effective they perceive these measures, (3) to report which support services horseracing staff report utilised, and (4) to determine the reported perceptions of horseracing staff on job security and personal well-being.

## 2. Materials and Methods

### 2.1. Design

A descriptive, cross-sectional, online survey design was used in this study. The use of online surveys allows interactions with a more diverse respondent group whilst obtaining a large sample at the convenience of the researcher and participant [30]. The survey was piloted using a purposeful sample (n = 20) of local horseracing staff.

### 2.2. Participants and Recruitment

Following institutional ethics approval by the XXXXXX (blinded for review) Human Research Ethics Committee (approval number ETHICS2019-55) and informed consent, eligible UK horseracing staff (*n =* 287) voluntarily provided useful unidentified online survey data. Participants were eligible if they were over 18 years old and were employed in the UK horseracing industry at the start of the UK lockdown (23 March 2020). This study considered racing employees as both those working directly with horses (grooms, trainers) and those working in administrative and organisation capacities (racecourse staff, racing secretaries) and categorised them into sectors for comparison (see Table 1).

Recruitment was achieved through personal and organisational industry contacts, collaborating partners and social media groups/pages to recruit participants who meet inclusion/exclusion criteria [31]. The sample was purposive and therefore not representative of the wider equestrian population. However, potential respondent bias was minimised by utilising a wide range of online sites to recruit participants [30].

### 2.3. Measures and Procedure

The online survey was conducted using Qualtrics CoreXM survey software. Participants completed 18 closed, and three open questions, which took approximately five minutes to complete. Questions were designed by the research team to investigate working practices in light of the current COVID-19 pandemic, and cover six areas of significance: employment status and job role, working practices during lockdown, changes to equine exercise (if applicable), employee support, perceptions of job security, and personal well-being (see File S1). Some working practice questions were adapted from previous literature into the racing industry [19,32].

Prior to starting the questionnaire, participants were given information pertaining to their data protection rights, risks and benefits, and withdrawal procedures before being asked to consent to the study; no personal data were collected. The survey was live from 20 April to 29 May 2020 and 89.9% of participants were obtained in the first 14 days; this data collection was inclusive of the first phase of lockdown [14].

### 2.4. Data Analysis

Data were exported from Qualtrics CoreXM (UK) to Microsoft Excel (Office 365, UK) and grouped according to industry sector, and by full- or part-time employment status, in order to enable the impact of COVID-19 by these characteristics to be evaluated. Frequency analysis assessed how coronavirus had impacted working conditions, hours, pay, and use of support services. Following assumption testing for normality, data were analysed using IMB Statistical Product and Service Solutions (SPSS) software version 26 (UK). Chi-squared tests for independence were used to identify associations; significance measured at *p* ≤ 0.05. Binary logistic regression (BLR) was undertaken to determine predictors for working during the pandemic within the horseracing industry. Univariate logistic regression was performed for all factors (Table 2) using the dichotomous variable “working or not working” to assess potential risk and inform model building. Factors were considered eligible for inclusion in the multivariate model if the level of significance was *p* ≤ 0.05 or if the removal of the factor had a significant impact on the model (*p* ≤ 0.05) [33].

Multivariate models were refined through a backward stepwise process with variables retained if likelihood ratio test *p* ≤ 0.05 [33]. As each factor was removed, the effect on the model was observed by checking the significance level of the Omnibus test (*p* ≤ 0.05) to ensure that any factors which exerted a significant effect on the model were not discarded [33]. The fit of each model was assessed using the Hosmer–Lemeshaw goodness of fit test [34] and the predictive ability of the model was examined through receiver operating characteristic (ROC) curve analysis [35].

Content analysis [36,37] was used for the three open-ended questions which looked at why staff were currently still working during lockdown (if applicable), the impact of lockdown restrictions on their working practices, and any recommendations for improvements. The first author familiarised themselves with the textual data and engaged in constant comparison to chunk the text into segments [38]. During the organisation phase, an inductive approach was used to generate and apply descriptive codes to the comments according to the content. Related codes were combined to produce subcategories and summarised into key categories based on the content [39]. This analysis was repeated across all three open-ended questions and evaluated by the remaining authors to ensure objectivity and rigor [38].

## 3. Results

A total of 287 participants fully completed the survey, comprising 63.1% (n = 181) full-time, 13.2% (n = 38) part-time and 20.6% (n = 59) self-employed staff. The remaining 3.1% (n = 9) selected option ‘other’, which consisted of casual, unemployed, those on sick leave and directors of limited companies. When asked, 53.7% (n = 154) of all staff were still working at the time of completing the survey. Employment sectors were broken down as per Table 1, with most staff working in either a racecourse-based role, or as a racing groom on a jump yard.

### 3.1. Working Practices

A significant association was found between racing sector and whether they were still working during the pandemic (χ^2^ (13, n = 287) = 47.211, *p* < 0.001). Flat racing grooms were twelve times more likely to be working (*p* < 0.001, 95% CI [3.43–46.23] Table 3), and breeding were staff sixteen times more likely to be working compared to jump racing grooms (*p* < 0.05, 95% CI [2.89–99.36]). When grouped by larger sectors, horse-related roles, administrative staff, and senior management, administrative staff were six times more likely to be working than other roles (*p* < 0.05, 95% CI [1.37–30.21]). In addition, those who did not have accommodation as part of their role in racing were 0.2 times less likely to be working compared to those in accommodation (*p* < 0.01, 95% CI [0.09–0.63]).

Of the 154 staff still working at the time of completing the survey, 94.8% (n = 146) answered a question about working hours. Of these, 54.1% (n = 79) of staff reported working the same hours as before, 32.8% (n = 48) reported working fewer hours and 13.0% (n = 19) reported working more hours than prior to the COVID-19 pandemic. A significant association was found between which sector staff worked in, and the hours they reported working during COVID-19 lockdown compared to previously (χ^2^ (26, n = 146) = 47.994, *p* < 0.01). Whilst most sectors appeared to be working the same, or fewer hours than prior to the pandemic, the breeding sector were more likely to report working more hours during lockdown.

Three themes were identified as reasons to still be working during the pandemic: horse care, administration and management responsibility. Horse care included daily care, stud work or exercise, and was identified 119 times. Administrative tasks, including planning for return to racing, employee support and working from home were all highlighted, a total of 33 times. Management responsibility was cited 17 times, and identifying seniority in roles at work, or boss/owner status as reasons to maintain working.

Of those 130 staff identified as not working at the time of the survey, when asked why they were not working, 42.6% responses identified furloughed employment status (n = 72). Reasons for not working are identified in Table 4—staff were able to select more than one answer for this question. Of those who selected ‘other’, staff highlighted casual employment status or sick leave as reasons.

### 3.2. Changes to the Workplace 

Of staff currently working, 146 chose to give answers to whether any changes were made at their workplace as a result of COVID-19. A total of 93.2% (n = 136) reported workplace changes made as a result of the pandemic. When asked what changes had been made, 422 responses were given, reporting additional hand washing and distancing measures as most common. All changes are reported in Table 5—participants were able to select more than one option when answering this question. Other included following Racing Stakeholder protocols, emergency veterinary visits only, digital paperwork and use of gloves.

**Figure 1:**
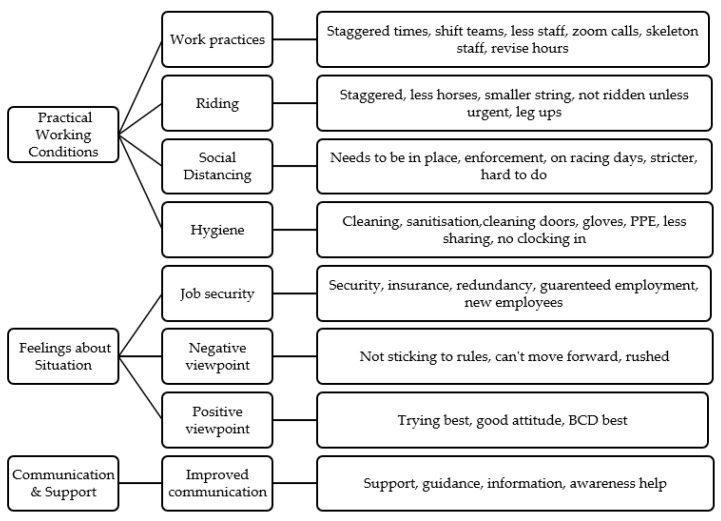
Recommendations made by staff (key themes).

When asked whether these changes were perceived as effective, 87.8% (n = 115) of staff reported changes as being positively effective (extremely, very and moderately effective). There was a significant association between racing sector and whether changes were deemed effective (χ^2^ (52, n = 131) = 92.996, *p* < 0.001). Flat racing staff were more likely to report changes as less effective than other racing sectors. Racing staff were asked about any recommendations to improve the current working conditions in the industry as an open-ended question. Three main themes were identified, surrounding practical/logistical recommendations, feelings about workplace and current changes, and communication (Figure 1).

### 3.3. Perceptions of Job Security 

All staff were asked about likelihood of job security in three months’ time, and 44.6% (n = 128) of staff felt their job was ‘probably secure’. Overall, 67.2% (n = 193) of participants answered positively towards security of their job (definitely or probably secure), whilst only 15% (n = 43) answered negatively, with a further 17.8% (n = 51) unsure about their job security in three months’ time. Perception of job security was significantly associated with whether an individual was currently working during the pandemic (χ^2^ (4, n = 287) = 37.980, *p* < 0.001). Perception of job security was found to predict likelihood of working (*p* < 0.001). Staff who selected unsure or probably not secure were 0.2 times less likely to be working than staff who selected definitely secure in their job (*p* < 0.01, 95% CI [0.08–0.56] and *p* < 0.05, 95% CI [0.08–0.88], respectively). Staff who felt their job security was definitely not secure after the pandemic were 0.08 times less likely to be working than staff who were definitely secure in their job (*p* < 0.01, 95% CI [0.01–0.42]).

Job security was significantly associated with which sector a racing employee worked in (χ^2^ (52, n = 287) = 75.653, *p* < 0.05). Whilst most staff felt positively towards job retention in three months’ time, those working in training yards and those working as a racehorse trainer were less confident of their job security. In addition, employment status was significantly associated with perceptions of job security (χ^2^ (12, n = 287) = 44.266, *p* < 0.001). Staff in full-time roles were more likely to select ‘definitely or probably’ secure, whilst staff employed part time, or self-employed were more likely to select less definitive options such as ‘probably’, ‘probably not’, or ‘unsure’.

### 3.4. Use of Support Services and Personal Well-Being 

**Figure 2:**
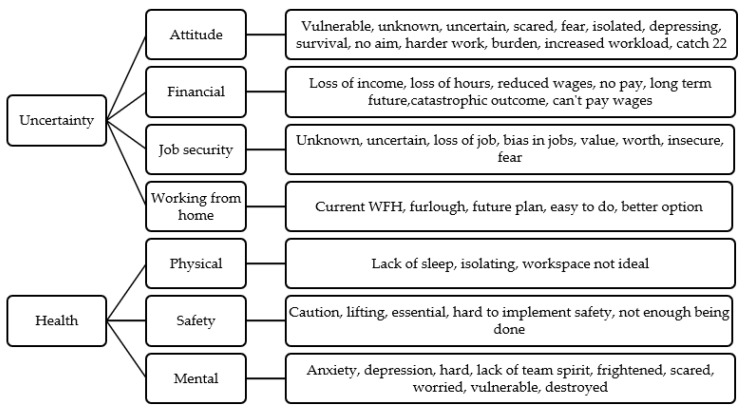
Further impacts of COVID-19 on staff (key themes).

A total of 259 people commented on use of support services. Whilst 70.66% of participants reported having not accessed any racing-specific support services online or via phone (n = 183), of those who had, the British Horseracing Authority (BHA), Racing Welfare and the National Trainers Federation (NTF) were reported as most used (10.42%, 4.63%, 4.63%, respectively). When asked about financial support services, 81.82% of participants reported not accessing any support services for financial purposes. The most common reported financial support was the self-employed government financial scheme (8.66%). Participants were asked about any further comment on how COVID-19 had impacted their working conditions. Whilst 85 responses reported no further comments, remaining data were analysed, resulting in the following themes (Figure 2):

## 4. Discussion

This study aimed to investigate the impact of COVID-19 on staff working practices across UK horseracing during the initial lockdown phases. The results found that approximately half of staff were still working during the pandemic, dependent on their sector and job role, and over 80% of staff reported that workplace changes prioritised staff health and safety. For training yards, the changes made to working practices may affect staff’s ability to successfully carry out daily tasks, which could negatively impact the welfare of the horses in their care. Perceptions of job security post-pandemic continues to be a concern, and further development of employee support structures to target concerns is advised.

### 4.1. Working Practices

Just over half the staff surveyed were still working at the time of completing this survey, and of those, most reported their working hours to be similar or less than prior to lockdown. Three themes were identified to explain why someone was still working during lockdown: responsibility for daily equine care [16], administrative responsibilities that were completed from home, or working in a senior or management position. Unexpected administrative tasks substantially increased during the initial lockdown phases, including alterations to work schedules, pay, furlough applications and additional risk assessments [1]. Those in managerial positions may be more likely to be working due to the high number of equine and racing businesses being classified as small- to medium-sized enterprises (SMEs) [1,40], thus resulting in managerial staff, directors and owners being required to continue working during the pandemic to cover staff, or opting to support the remaining workforce to reduce labour costs within the organisation. Understanding the reasons for maintaining work during the pandemic is important when reflecting on additional support mechanisms that may be required for staff moving forwards, and in identifying target groups for preventative strategies in the event of a second wave.

Racing sector was a significant predictor for working during the pandemic; when grouped by type of role, administrative staff were more likely to be working than stable staff, due to following work from home (WFH) protocols, whilst maintaining mitigation measures [41]. Flat racing grooms and stud grooms were more likely to be working than other stable staff. The timing of lockdown coincided with the thoroughbred foaling season, resulting in staff working on studs being busier during the lockdown period [42]. Flat grooms were preparing for the resumption of racing during the initial lockdown phases, proposed to return first in the calendar [1]. The delay for jump racing resulted in most jump yards roughing off horses, reducing the workload required and enabling them to reduce staff numbers on site, whilst maintaining high standards of equine welfare.

### 4.2. Changes to Work Health and Safety

Nearly all staff reported changes to their working practices as a result of COVID-19, with most staff viewing these changes as effective. The racing sector has previous experience with extensive equine disease protocols, as evidenced by the equine flu outbreak in 2019. Rapid changes to racing protocol, additional equine distancing measures and extra precautionary checks (such as additional vaccination and temperature checks) [3] meant that most racing sectors had previously experienced reactive alterations to practice based on equine welfare and were therefore equipped to quickly adapt similar strategies for staff.

Cooperation with change improves substantially by transparent and regular communication, which was a priority of the COVID-19 Steering Group and may explain why most staff reported being happy with the changes made to their working environments [43]. Some text comments requested more information from organisations, which at the time of the survey was not unsurprising as the government had not approved the resumption of racing plan, leaving many feeling uncertain of their future, or the length of the lockdown. Further research is recommended to determine whether changes in perception of communication has altered since the resumption of racing.

Although not directly investigated in this study, it would be interesting to investigate whether those racing organisations where managerial staff remained in active and visible roles during the pandemic saw a more positive perception of crisis management and handling of the pandemic situation than those with less visible management. Workers often look to leaders for example, particularly when large change and collective action is required [43,44]. Visible management staff may help to coordinate individuals and promotes public messages of safety, such as mitigation and hygiene measures in cases of disease outbreak [44]. Management who are clearly visible to lower level staff during these times are more positively received thus resulting in increased adherence to behavioural change in their team, greater self-efficacy and increased ‘buy in’ to changes made.

The most common methods of change were increased distancing measures, additional handwashing requirements (and therefore increased facilities made available) and the reduction in staff onsite at any one time, which are the most common mitigation measures identified by research and the government [1,10,14,23]. Distancing measures, alongside additional hygiene considerations in the workplace, have been shown to reduce the transmission of COVID-19 [9]. However, as with any community-based strategy, the success of these changes rely on individual adherence [10] and significant changes in group and individual behaviour [44]. Behaviour is significantly influenced by social norms, and group behaviour is key to ensuring individuals stick to changes made [44]. Employees within the racing industry have been previously reported to regulate emotional displays to meet the organisation’s expectations of the role [22], which is suggested to create an organisational culture where the employees act, think and feel in accordance to expectations, and new staff entering are taught to adhere to these cultural norms [45]. This may explain the staff ‘buy in’ to specific COVID-19 changes, as the already established organisation culture encourages staff to adhere and follow certain social guidelines within the industry, including the addition of safety protocols.

There was some concern regarding flat racing stable staff, who were more likely to report the COVID-19 changes as less effective than other sectors. Due to the uncertainty of returning to racing and the need to keep horses in training, flat training yards were more likely to be busier during this period, which could result in difficulties adhering to social distancing practices. However, staff working in the breeding sector were also busy due to the timing of the foaling season, and did not report negative perceptions of the changes made at their establishments as seen by flat racing grooms, suggesting that the higher activity levels on the yard are not the only reason flat racing staff may have perceived concerns over the efficacy of COVID-19 changes. As an example, the Thoroughbred Breeding Association (TBA) did provide extensive COVID-19 Employee Training Guidance to all breeding establishments, which outlines mitigation measures for individual activities whilst working on a stud yard [42]. Whilst subsequent COVID-19 online courses have been developed for the wider racing industry, and NARS have produced significant guidance to support stable staff, the breakdown of mitigation measures by type of daily activity (such as horse handling, tractor driving, use of break rooms/shared kitchens) provided by the TBA could be considered best practice and may have enhanced the effectiveness of the changes made by the breeding sector [43,44]. The use of itemised and adapted mitigation measures for daily workplace activity from the TBA guidelines should be shared with the wider sector with the aim to support development of risk assessments for employers and where appropriate support staff in feeling safer at work.

Research on essential workers during lockdown highlighted that numerous staff were concerned about personal safety at work, mitigation measures and personal protective equipment (PPE) use [25], which was echoed by racing staff in text comments during this survey. Staff reported a concern for safety, health worries and an increased need to focus on risk assessment for tasks that may often require more than one person (thus breaking the 2 m distancing restrictions). An earlier lockdown survey conducted with only stable staff highlighted some concern regarding safety at work, with 36% of staff suggesting that they did not feel safe at work [23], whilst wider UK labour industries suggest that 1 in 10 staff are currently concerned about health and safety at work during the pandemic [14]. Whilst quantitative measures suggested that most staff found the changes at their workplace to be effective in enhancing their personal health and safety, this should be investigated further by individual employers to ensure that all necessary actions are being taken to maximise feelings of safety, particularly due to the hands on nature of numerous sectors within the racing industry.

Related to this, concerns regarding contact during mounting horses was highlighted, where staff are often required to give each other a ‘leg up’ to support getting onto the horse, as mounting blocks are anecdotally used less in racing compared to equestrian sports. This action requires significant contact between individuals and cannot be achieved whilst maintaining a 2 m social distancing rule. However, alternatives for leg ups are limited, as research shows that mounting from the ground (which can be done without assistance) is harmful to the horses’ welfare, and significantly associated with increased back pain [46]. The welfare of the horses is paramount in racing, and thus certain activities such as mounting using a leg up, may not be adaptable to the current virus mitigation measures. Where 2 m mitigation measures cannot be undertaken, 1 m restrictions with risk mitigation (such as masks and gloves) is advised by the UK Government for those working outdoors [14]. This is now being seen at racecourses since the return to racing and should be considered best practice for those working on training yards when mounting. It may also be beneficial to consider a review of effective social distancing measures for training yards now racing has resumed, with full staffing capacities and more horses now in full exercise. This may aid to further reduce any safety concerns for those working in yards and support staff who are riding out, whilst prioritising the health of the racehorse.

### 4.3. Perceptions of Job Security

The racing industry appear to be mostly positive when asked about their perceptions of job security in three months’ time. This may be due to the attachment of the ‘essential worker’ title to several roles within the sector which may have previously been seen as ‘lower-skilled roles’ due to lack of higher qualifications required to meet the demands of the roles, such as for racing grooms. Whilst racing grooms are classified by industry as a highly skilled, multifaceted job role, which requires significant knowledge and skill, negative external viewpoints of working with horses have been reported [4]. Spurk and Straub [47] identified that labelling roles as ‘essential worker’ resulted in a prestige boost for industries previously viewed as ‘unskilled labour’, which resulted in increases in job satisfaction and job commitment for employees in those sectors. Despite the quantitative results suggesting a more positive outlook, uncertainty about job security was a key theme emerging from several open-ended questions. Research indicates that anxiety over potential unemployment is a significant stressor during times of economic crisis, or previous disease outbreaks [25,48,49]. Prior to the pandemic, unemployment figures in the UK stood at 3.9% (Q1 data). However, predictions of a 6.1% spike in Q2 data are being proposed by economists due to a reduction in business and trade in the UK during lockdown [50]. An increase in unemployment is not unexpected; in 2007 (pre-recession), unemployment was 5.2% compared to a 21st-century peak of 8.5% in 2011 after the credit crisis [50]. A previous industry survey of only stable staff reported that 46% were worried about their job in the near future [23]. This study identified that more staff in training yards, and trainers themselves were worried about their jobs compared to wider sectors, such as staff working at racecourses, echoing the earlier research by NARS. This is likely as a result of trainers’ reliance on owners, horses and successful racing results in order to maintain business and therefore continue to employ grooms working in these yards. Those in less secure roles, such as part-time staff or self-employed trainers, were also more concerned about the impact of COVID-19 on job security. The International Labour Organisation (ILO) state that unprotected workers, including casual or self-employed staff, are likely to be disproportionally impacted by COVID-19 due to lack of sick leave, and limited options for income protection [51].

Job security and employee retention also pose substantial implications for the quality of care of racehorses on training yards following the lockdown [15,20]. As the industry has continued to expand, staff are increasingly expected to care for additional horses, with reports suggesting care of up to 5–6 horses, compared to the previous 2–3 per individual [15]. The increased workload on individual staff to care for an increasing number of horses also puts additional stress on the workforce when one or two members of the team are absent, unwell or leave. Continuous staff turnover negatively impacts the cyclical nature of teaching and skills sharing seen within the racing industry, reducing the dissemination of invaluable skills and experiences to younger generations [20]. The spread of misinformation is more prevalent when a workforce is unstable, as health and safety protocols and practices are often not relayed as efficiently [52]. Filby et al. [53] suggested that the risks found in working with horses are only mitigated by experienced staff. Within the racing sector, poor staff retention in training yards may negatively impact the communication of health and safety protocols as well as specific equine care and management practices to new or younger staff. Whilst already a wider issue (see Juckes et al. [20] for full review), the increased demands experienced by staff during and following the COVID-19 lockdown may increase the risk of workforce instability. The continuation and further development of employee support structures, such as helplines, additional workshops for employees, and financial support services, to promote job security within the industry following the pandemic are a necessity. In addition, further research should consider the medium to long-term effects of lockdown on racing staff with follow-up studies recommended to determine the impact on perceptions of job security in 3–6 months’ time, and 12 months’ time, and the possible wider implications on staff information sharing in the workplace and subsequent standards of equine welfare.

### 4.4. Use of Support Services and Personal Well-Being

Most staff reported not using support services available from the racing industry during the pandemic, but of those who did, the British Horseracing Authority (BHA), Racing Welfare, and the National Trainers Federation (NTF) were most common. After the lockdown announcement, the COVID-19 Steering Group created guidance for all staff required to work during the pandemic, including social distancing, equine specific welfare measures, and staff support opportunities. Due to the timing of the survey (end of April release), central guidance was already published to the sector, thus reducing the likelihood of staff reporting a need to further look for COVID-19-specific guidance. This study’s recommendations would be for the racing industry to continue to deliver COVID-19 information to all sectors of the industry through national organisations, unions and charities, as this approach was positively received by staff at the start of lockdown.

Most staff reported not accessing any financial help, from racing or the government schemes. Of those who did, the most commonly utilised support was the self-employed income support scheme offered by the government, which has received over 2 m claims and issued over £6.1bn in grants (correct at 17 May 2020). Holmes et al. [54] reported that more people were currently concerned with practical and logistical measures related to social distancing than financial worries. However, this may not account for long-term considerations of financial security. Economic welfare and growth are positively related to population health [55] and the effects of the lockdown will be far reaching. Previous public health outbreaks have resulted in interruptions to production lines, with global supply chains disrupted due to travel restrictions [55]. In addition, changes in buyer behaviour due to necessity or fear can impact labour supply decisions, whilst sickness within the workforce affects business costs and labour efficiency [55].

Experiences of uncertainty and concerns over personal physical and mental health were themes identified by staff during the pandemic, irrespective of whether that individual was actively working. Fear and anxiety during a global pandemic are expected, with the psychological effect suggested to be akin to terrorism or biological warfare threats [54]. Societal fear responses were seen during the 1918 Spanish Flu and the 2003 SARs-CoV outbreaks, with changes in consumer buying behaviour and increases in depression, anxiety and suicide reported during and post-outbreaks [54,55]. Government reports suggest increasing concerns about mental health during the pandemic [14], with more people worried about psychological and social effects, than fear of becoming ill, explaining the reduced focus on catching COVID-19 reported here [54].

Changes in working arrangements, such as work schedules, limited support and increased work demands, can also result in psychological distress [25]. Key themes identify a need for increased communication, and the effect of increased demands for healthy workers. An increase in demands placed on staff already under psychological distress from mitigation measures, and anxiety over the pandemic can result in burnout, reduced sleep, fatigue and physical ill health, affecting both staff efficiency and yard management standards [25]. Whilst the majority of concerns are based around personal responsibility hygiene practices, employers should carefully consider whether reducing the number of staff promotes increased physical demand on remaining employees, such as additional weight carrying requirements, which could result in an increased risk of injury [55,56,57,58]. Additional demands should be factored into risk assessments and counteracted by additional rest breaks, and preventative injury management strategies. Racing already reports a culture of ‘working whilst broken’, due to a fear of being irreplaceable [16] or lack of available staff cover [22,32]. The inability to work at normal capacity, due to injury, burnout or fatigue, may also increase the risk of injury to horses under the care of stable staff, or lead to poor management practices [15,16,19]. Research in the veterinary sector has identified that increased work stress, such as would be experienced during a pandemic, is a predictor of accidents in the workplace [59]. Lack of concentration, physical fatigue and burnout resulting from a stressful working environment or reduced mental resilience can negatively impact task efficiency by affecting visual acuity, accuracy and individual reaction time [57]. Slower reactions, or loss of focus around horses could result in preventable injury to both parties or result in subpar management and care of the horse, such as errors in feed rationing [20,22]. Previous research identified areas of concern around employee mental health in the racing sector [32], and wider research supports that global pandemic situations can result in increased risk of depression, anxiety and decreased quality of life [25]. Future research to further investigate how continuous lockdown and racing restrictions have affected staff well-being, and the perceptions of senior management on daily workforce productivity and standards would be advised by the authors. Working from home is now a more widespread phenomenon as a mitigation measure during lockdown. However, research suggests that this can result in both positive and negative reactions from staff [41]. Whilst most staff in this survey who were working from home were positive about their arrangements, there were some concerns highlighted regarding the efficacy of that environment, including workspace limitations and home/work balance being impacted. The British Psychological Society (BPS) developed a psychological approach for employees and employers which could be utilised within the racing sector to support staff in ongoing work from home situations. The SHARE approach is designed to facilitate communication, adaptation and flexibility and outlines practical solutions for both parties to maximise well-being and productivity [41]. This paper has adapted these guidelines to include racing-specific recommendations (Table 6).

There are limitations to consider within the study. The online sample, although a quick way to obtain access to a wider population of staff, may have been subject to self-selection bias [60]. This may have skewed the participants to include only staff who were significantly impacted by the lockdown. The study’s largest sector groups were from grooms and racecourse staff, which may skew the data, and wider generalisation should be considered with caution.

## 5. Conclusions

The findings of this study suggest that most of the racing industry positively received changes made by the sector and employers to tackle the pandemic, and for staff still working during lockdown, their health and safety was prioritised. The racing sector’s ability to swiftly and successfully develop COVID-19 protocols to protect staff has led to positive feedback on the efficacy of these protocols and should be commended. The continuation of COVID-19 information and staff guidance through national organisations, unions and charities is advised as this approach was positively received at the start of lockdown. There is some staff concern for future job security, and this should be a key area of focus for the industry moving forwards to ensure a stable workforce. Stability in the workforce ensures higher standards of care for the horse, as health and safety protocols, and knowledge of individual horse’s requirements are less likely to be become diluted through continuous staff turnover. Further development of employee support structures, such as helplines, additional employee workshops, and financial support services to promote job security within the industry, following the pandemic are also advised.

## Figures and Tables

**Table 1:** Staff working in different sectors of the racing industry.

Racing Sector	N	Percentage Total (%)
Training Yard (jump)	58	20.2
Training Yard (flat)	31	10.8
Training Yard (mixed)	44	15.3
Breeding Sector	18	6.3
Jockey	8	2.8
Trainer	10	3.5
Point to Point	4	1.4
Sales	4	1.4
Pre-Training	3	1.0
Racecourse	60	20.9
Racing Administrator	21	7.3
Coaching and Education	4	1.4
Media and Communications	10	3.5
Other	12	4.2

**Table 2:** Factors included in the univariate model.

Factor	Description	Categories
Grouped Sector	Sector grouped by reason for working, coded from question 7 (see File S1).	Categorical Variable: stable staff (equine responsibility) (reference category), administrative role, management role.
Sector	10 racing employment sectors previously identified by prior industry research [5,6,32].	Categorical Variable: training yard (jump) (reference category), training yard (flat), training yard (mixed), breeding, jockey, trainer, racecourse, racing administration, coaching and education, media and communications, other, pre-training, point to point, sales.
Employment Status	Status of employment at the onset of initial government lockdown phase.	Categorical Variable: full time (reference category), part time, self-employed, other.
Perceived Job Security	How confident an employee felt regarding their job security in three months’ time.	Categorical Variable: definitely secure (reference category), probably secure, unsure, probably not secure, definitely not secure.
Accommodation	Whether accommodation was provided by their racing-specific employer.	Categorical Variable: yes (reference category), no, not applicable.

**Table 3:** Final BLR multivariate model (C1).

Model C1: Working Backwards Stepwise (LR)	Total N = 284	*p* Value	Odds Ratio	95% Confidence Interval	B Value
	N–per Category				
Grouped sector:		0.059			
Stable staff	164	REF			
Admin staff	35	0.018	6.438	1.372–30.218	1.862
Senior management	85	0.533	2.074	0.120–20.520	0.729
Sector:		0.003			
Jump racing groom	57	REF			
Flat racing groom	31	0.000	12.606	3.437–46.228	2.534
Mixed training yard	44	0.034	2.752	1.082–7.003	1.012
Breeding	18	0.002	16.946	2.890–99.367	2.830
Jockey	7	0.148	4.096	0.607–27.615	1.410
Trainer	10	0.122	8.740	0.561–136.051	2.168
Racecourse	59	0.765	0.710	0.075–6.708	−0.342
Administration	21	0.434	0.514	0.097–2.726	−0.665
Coaching/Education	4	0.839	0.744	0.051–10.971	−0.295
Media/Communications	10	---	---	---	---
Other	12	0.772	0.694	0.059–8.223	−0.365
Pre-training	3	0.454	2.902	0.178–47.253	1.065
Point to point	4	---	---	---	---
Sales	4	0.999	2705340068.707	0.000	21.718
Job security:		0.000			
Definitely secure	64	REF			
Probably secure	127	1.000	1.000	0.480–2.082	0.000
Unsure	50	0.002	0.215	0.083–0.558	−1.536
Probably not secure	24	0.030	0.274	0.085–0.882	−1.294
Definitely not secure	19	0.002	0.084	0.084–0.418	−2.471
Accommodation:		0.014			
Yes	44	REF			
No	210	0.004	0.248	0.097–0.633	−1.395
Not applicable	30	0.082	0.301	0.078–1.165	−1.200

**Table 4:** Reasons for not working during lockdown.

Reason for Not Working	N	Percentage (%)
Furlough	72	42.6
Made redundant	8	4.7
Volunteered not to work	5	8.5
Self-isolating due to personal illness	1	0.6
Self-isolating due to family illness	1	0.6
No horses in training	28	16.6
No racing	47	27.8
Other	6	3.6

**Table 5:** Common workplace changes made as a result of the pandemic.

Changes Made	N	Percentage (%)
Antibacterial or handwashing facilities made available	94	22.3
Limiting number of staff working at any one time	80	19.0
Distancing measures enforced	97	23.0
Limiting use of shared resources/equipment	60	14.2
Additional cleaning of shared resources/equipment	60	14.2
Working from home	23	5.5
Other	10	2.4

**Table 6:** SHARE key guidelines for employers and employees, adapted to the racing industry (adapted from Kinman et al. [41]).

SHARE	Employers	Employees
Safe homeworking	Consider your duty of care Provide practical guidance to employees Maintain trust and communication	Identify an appropriate workspace Plan your day and schedule breaks Consider privacy and data regulations
Help yourself and others	Set realistic expectations Communicate and check in regularly with employees Support the development of digital resilience skills (Racing2Learn) Consider employee’s hidden costs	Communicate and stick to your schedule Make sure to switch off regularly Develop new skills if possible, including skills for digital resilience (Racing2Learn)
Adapt to change	Recognise diverse needs and circumstances Understand the risks Assess and address risk	Allow time to develop your own style of homeworking and establish a routine Set boundaries between home and work life Stay socially connected and consider the positives
Relieve the pressure	Use a flexible approach Show support Role model healthy behaviours	Maintain work-life balance Keep active Sleep well, eat well
Evaluate	Regularly review your SHARE approach with each employee	Regularly review your SHARE approach with your manager

## Supplementary Materials

The following are available online at https://www.mdpi.com/2076-2615/10/11/2003/s1, File S1: Questionnaire.
