# Supplementary material for: The Impact of COVID-19 on Staff Working Practices in UK Horseracing
Source: Animals (Basel). 2020 Oct 30;10(11):2003. doi: 10.3390/ani10112003 (PMC7692747; doi:10.3390/ani10112003)
Supplement: Supplementary file 1 [file animals-10-02003-s001.pdf]

Questionnaire

☐ Q1. I agree to the terms & conditions of the study, outlined in the pre-questionnaire information above

Q2. Which sector of the racing industry best describes where you work?

○ Training Yard (jump)<br>○	○ Jockey	○ Coaching and Education
○ Training Yard (flat)	○ Trainer	○ Media and Communications
○ Training Yard (mixed)	○ Racecourse<br>○	○ Other, please state ______________________________
○ Breeding sector	○ Racing Administration	

Q3 Please select your current employment status.

- ○ Full time
- ○ Part time
- ○ Self-employed
- ○ Other, please state ____

Q4 As a result of Covid-19, how secure do you feel your current role is in industry? (i.e. you would still be employed in 3 months' time if you so wished to remain)

Definitely secure	Probably secure	Unsure	Probably not secure	Definitely not secure

Q5 Are you currently living in accommodation provided by your employer, as part of your role in racing?

○ Yes

○ No

○ Not applicable

End of Block: Demographics

Start of Block: Working

Q6 Are you still actively working at the time of completing this survey?

○ Yes

○ No

Skip To: End of Block If Q6 = No

Q7 Please give the reasons you are currently still working e.g. essential worker, horses need care etc.

____

____

____

____

____

Q8 How has the Covid-19 pandemic impacted the hours you can work

○ I am working fewer hours than before

○ I am working the same hours as before

○ I am working more hours than before

Q9 Have there been any changes to your work environment since the pandemic?

○ Major Changes

○ Minor Changes

○ No Changes

Skip To: End of Block If Q9 = No Changes

Q10 What non-riding changes have been made? Please select all that apply.

- ▢ Additional anti-bacterial/hand washing resources available
- ▢ Limited number of staff working
- ▢ Distancing measures
- ▢ Limiting use of shared resources/equipment
- ▢ Additional cleaning of equipment/shared resources
- ▢ Other, please state ____

Q11 Do you feel these non-riding changes are effective?

- ○ Extremely effective
- ○ Very effective
- ○ Moderately effective
- ○ Slightly effective
- ○ Not effective at all

End of Block: Working

Start of Block: Horses

Q12 Are horses still being ridden out at your establishment?

- ○ Yes
- ○ No
- ○ Not applicable to my sector

Skip To: End of Block If Q12 = No

Skip To: End of Block If Q12 = Not applicable to my sector

Q13 What measures have been taken to facilitate social distancing when riding out? Please select all that apply.

- ▢ Fewer horses per slot
- ▢ Slower work being undertaken
- ▢ Tack cleaned before & after every use
- ▢ Tack cleaned daily
- ▢ Only certain horses currently in work
- ▢ No measures have been taken
- ▢ Other, please state ____

Q14 Do you feel these riding changes are effective?

Extremely effective	Very effective	Moderately effective	Slightly effective	Not effective at all

End of Block: Horses

Start of Block: Not Working

Display This Question:

If Q6 = No

Q15 Please give the reasons you are not currently working. Please select all that apply.

- ▢ Temporary leave of absence from work - forced
- ▢ Made redundant
- ▢ Volunteered not to work
- ▢ Self-isolation (you are ill)
- ▢ Self-isolation (family member/dependent ill)
- ▢ No horses in training
- ▢ No racing
- ▢ Other, please state ____

Display This Question:

If Q6 = No

Q16 If you are not working, are you still being paid?

- ○ Yes - 100% of my salary
- ○ Yes - 80% from government schemes
- ○ Less than 80%, but some
- ○ No, I am not being paid

End of Block: Not Working

Start of Block: Support

Q17 Have you accessed any of the following services for support or advice (online or phone) during the Covid-19 pandemic? Please select all you have personally accessed.

- ▢ Racing Welfare
- ▢ Racing's Occupational Health
- ▢ National Association of Racing Staff (NARS)
- ▢ Injured Jockeys Fund (IJF)
- ▢ Professional Jockeys Association (PJA)
- ▢ National Trainers Federation (NTF)
- ▢ British Horseracing Authority (BHA)
- ▢ Other, please state ____
- ▢ I have not used any support services

Q18 Have you applied for any of the following FINANCIAL support services during the Covid-19 pandemic? Please select all you have personally applied for.

- ▢ Racing Welfare Hardship grants
- ▢ Government financial support (employed)
- ▢ Government financial support (self-employed)
- ▢ Other, please state ____
- ▢ I have not used any support services

Q19 What suggestions, if any, do you have for amending current working practice in the horse racing industry during the Covid-19 pandemic?

____

____

____

Q20 If you have any other comments about how Covid-19 has affected your working conditions, please outline them here

____

____

____

End of Block: Support
